# How Big Are the Waves

**Published:** 1888-04

**Authors:** 


### HOW BIG ARE THE WAVES?

### EXAGGERATED NOTIONS AS TO THE TURBULENCY OF THE STORM SWEPT SEA.

It is a very common phrase to speak of the waves during a storm as "running mountains high;" but this really means nothing. Accurate measurements made by Scoresby proved that, during storms, waves in the Atlantic rarely exceed 43 feet from hollow to crest, the distance between the crests being 560 feet, and their speed 32½ miles an hour. More recent observations in the Atlantic give from 44 feet to 48 feet as the highest measured waves; but such heights are rarely reached, and, indeed, waves exceeding 30 feet are very seldom encountered. The monsoon waves at Kurrachee breakwater works were found to dash over the wall to the depth of 18 feet, or about 40 feet above mean sea level.

The greatest heights of waves on the British coast were those observed in Wick Bay—so famous for the exceptionally heavy seas which roll into it—being 37½ to 40 feet. Green seas to the depth of 25 feet poured over the parapet of the breakwater at intervals of from seven to ten minutes, each wave, it was estimated, being a mass of 40,000 tons of water, and this continuously for three days and nights. The iron pile lighthouse erected on the Bishop Rock was carried away by unbroken seas striking the dwelling, the floor of which was 85 feet above high water. A tower of granite was subsequently erected on the Bishop, and in 1860, the waves carried away the fog bell, weighing 3 cwt., at an elevation of 100 feet above the sea. In the Shetland Islands blocks of stone have been quarried at the height of 70 to 75 feet above the mean sea level.

But these instances of the action of the waves during storms sink into insignificance when it is mentioned that blocks of concrete, weighing 1,350 to 2,600 pounds respectively, were carried away by the repeated assaults of the wild rollers of Wick Bay. The depth to which wave action extends has been differently estimated, but it varies from 70 to 150 feet, as shell fish which are known only to live at these depths are thrown upon the shore during heavy gales, and it has been ascertained that shingle is moved in a depth of 50 feet. For all practical purposes, however, so far as harbor works are concerned, it is found that there is little movement of materials under 18 to 20 feet below water, the foundations of breakwaters hitherto constructed not having been disturbed below these levels.—*Scotsman.*

